# Increased Serum Levels of Dickkopf‐1 and Sclerostin as WNT Signaling Pathway Inhibitors in Celiac Disease Patients

**DOI:** 10.1002/iid3.70458

**Published:** 2026-05-13

**Authors:** Mohammed Nibras Thabit Al‐Ani, Saad Kadhim Taher Yahya, Majid Dastorani, Hanie Mirkarimi, Sima Besharat, Marie Saghaeian Jazi

**Affiliations:** ^1^ Metabolic Disorders Research Center, Biomedical Research Institute Golestan University of Medical Sciences Gorgan Iran; ^2^ Golestan Research Center of Gastroenterology and Hepatology, Jorjani Clinical Sciences Research Institute Golestan University of Medical Sciences Gorgan Iran

**Keywords:** celiac disease, Dickkopf‐1, parathyroid hormone, sclerostin, villus atrophy, WNT signaling pathway

## Abstract

**Background:**

Celiac disease (CD) is an autoimmune enteropathy. The WNT signaling pathway acts in adult intestinal epithelium maintenance.

**Aim:**

To evaluate the level of two WNT antagonists, including dikkopf‐1 (Dkk‐1) and sclerostin, in CD patients compared to controls.

**Methods:**

A total of 43 cases and 45 controls were enrolled in this case‐control study. The serum levels of Dkk‐1, sclerostin, citrulline, and parathyroid hormone (PTH) were measured using the ELISA method. Moreover, the levels of vitamin D3 were measured using the HPLC method.

**Result:**

Our findings illustrated an elevation of both WNT antagonists (Dkk‐1, *p* < 0.0001 and sclerostin, *p* = 0.002) in CD patients compared to controls. Moreover, the levels of PTH (*p* < 0.0001) and Ca (*p* = 0.009) were higher in CD patients. The level of citrullin was directly correlated with sclerostin (*R* = 0.71), PTH (*R* = 0.53), and Dkk‐1 (*R* = 0.29). ROC curve analysis indicated that serum levels of Dkk‐1 (AUC = 0.83), sclerostin (AUC = 0.7) and, PTH (AUC = 0.91) can differentiate CD from control.

**Conclusion:**

Celiac patients have increased levels of the Dkk‐1 and sclerostin, together with PTH as bone modulators, correlated with citrulline level, known as a potential marker for enterocyte mass. These results collectively indicate the significance of Dkk‐1 and sclerostin in the development of celiac disease; however, further research is needed to explore their possible diagnostic and therapeutic uses in the future.

## Graphical abstract

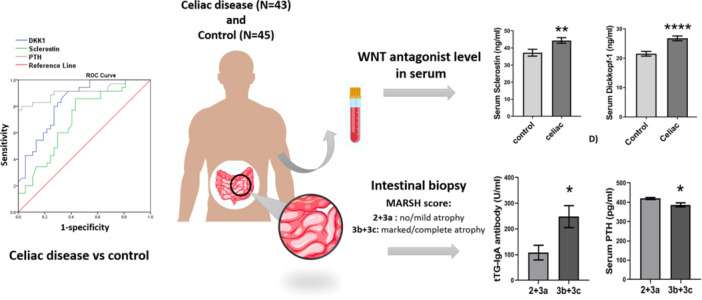

## 1 Introduction

Celiac disease (CD) is an autoimmune disorder triggered by the ingestion of gluten in genetically predisposed individuals, characterized by duodenal villous atrophy and intraepithelial lymphocytosis, leading to malabsorption and gastrointestinal as well as extraintestinal symptoms [1]. CD occurs up to 1% globally with a higher incidence in women, however, there is variation between different regions [2, 3]. For example, data reported in Iran show a prevalence of 3% (95% CI: 0.03–0.03) according to the serology and 2% (95% CI: 0.01–0.02) based on biopsy‐confirmed CD [4]. More recently, an epidemiological study reported even higher prevalence of celiac in western Iran (the 3‐year prevalence of 5.49/100,000 population) [5].

The real incidence of celiac disease has been increasing in recent decades and the reason might be related to environmental factors that may promote loss of tolerance to dietary gluten [6]. Currently, the only available treatment for the condition is a strict, life‐long gluten‐free diet [7]. Early diagnosis of celiac disease is important to prevent nutritional deficiency and long‐term risk of gastrointestinal malignancy. Current diagnosis of celiac disease depends on clinicopathological correlation: history, presence of anti‐transglutaminase antibodies, and disease confirmation by intestinal biopsy and duodenal tissue damage evaluation measured by MARSH score [8].

As reported previously, the intestinal stem cell (ISC) plays a critical role in epithelial regeneration after ionizing radiation induced gut injury [9]. Different mechanisms including the WNT/β‐catenin signaling pathway are necessary for self‐renewal and maintenance of the intestinal epithelium. Much evidence supports the crucial role of WNT signaling in the regulation of ISC function, and the maintenance of epithelial architecture [10]. Regarding the fundamental role of the WNT signaling pathway in the intestinal epithelial maintenance, here in the current study, we hypothesized the possible function of WNT regulators in celiac patients which are characterized with intestinal damage.

The WNT/β‐catenin signaling pathway is a highly conserved and tightly controlled molecular mechanism that regulates embryonic development, cellular proliferation, and differentiation [11]. WNT signaling can be regulated by multiple mechanisms including different WNT ligands, agonists, and antagonists interacting with the receptors. Dickkopf (Dkk) is the name of a protein family in the vertebrates, consisting of 4 members, named Dkk‐1–4, varying in size between 255 and 350 amino acids [12]. Dkk‐1 is an inhibitor of the WNT pathway and platelets represent a major source of Dkk‐1 in humans [13]. Dkk‐1 is induced by inflammatory cytokines during colitis and exacerbates tissue damage by promoting apoptosis of epithelial cells. However, little is known about the physiologic role of Dkk‐1 in normal intestinal homeostasis and during wound repair following mucosal injury [14]. Indeed, DKK‐1 has been reported to modulate immune responses through leukocyte infiltration, polarization of T cells to T helper 2, resulting in Th2 cell cytokine production in a murine model of allergen challenge and *Leishmania* (L.) major infection [15].

Another WNT antagonist is Sclerostin, a human bone tissue protein encoded by the *SOST* gene. Sclerostin belongs to the bone morphogenetic protein family of antagonists and is involved in the antianabolic processes of bone formation [16]. Sclerostin inhibits the functions, differentiation, and survival rates of osteoblasts, as it promotes the apoptosis of these cells. By binding to low‐density lipoprotein receptor‐related protein 5/6 receptors (LRP‐5/6), this protein blocks the Wingless‐type mouse mammary virus integration site (WNT) signaling pathway in osteoblasts [17]. Therefore, sclerostin has a pivotal role in bone biology and turnover. In animal models, SOST depletion not only affects B cell development in the BM relatively early but also creates an inflammatory bone marrow microenvironment that may become more severe over time [18]. Moreover, sclerostin‐deficient mice showed enhanced B cell response to T‐independent antigens, however, responses to T‐dependent antigens were impaired indicating that sclerostin can also affect peripheral B cell immune response in animal model [19].

The mean level of sclerostin has been reported to be decreased in systemic autoimmune diseases (nonsignificant), including Crohn's disease patients [20]. Moreover, in inflammatory bowel disease (IBD) patients with axial spondyloarthritis, the level of sclerostin (SOST) is decreased, but the level of anti‐SOST‐IgG is elevated compared to patients with only peripheral arthritis, IBD, and controls [21].

Both Sclerostin and DKK‐1, the two WNT antagonists, have shown to have role in immune system modulation beyond their primary function in bone regulation. Emerging evidence supports sclerostin function as immune regulator in B cell development and B cell immune response [18, 19], and DKK‐1 role in Th‐2 cytokine production [15]. Considering WNT antagonists (DKK‐1 and sclerostin) immune‐regulatory function as well as the pivotal role of WNT signaling in the intestinal epithelium homeostasis and crypt repair, thus in this study, we postulated that the level of Dkk‐1 or sclerostin may be associated with CD patients intestinal mucus atrophy or auto‐inflammation. Indeed, considering the well‐known modulatory function of WNT antagonists in bone remodeling, the biochemical factors of bone metabolism (PTH, Ca, and vitamin D3) were also investigated in the current study.

## 2 Material and Methods

### 2.1 Sampling

This study was a case‐control study and the samples (43 cases and 45 controls) were available from a previous project (IR.GOUMS.REC.1398.164). The samples were collected from the Golestan Research Center of Gastroenterology and Hepatology, Sayyad Shirazi Hospital, Gorgan, Iran. The inclusion criteria for cases were celiac disease diagnosed and confirmed with duodenum biopsy aged 20–65 years. The control group included from individuals who were referred to the endoscopy center of Sayyad Shirazi Hospital in Gorgan for other reasons and the celiac was ruled out by duodenal biopsy. All individuals with IgA deficiency were excluded from the current study. Both groups were matched in terms of age and sex (*p* > 0.05, Table 1). Informed consent was obtained from each patient included in the study and the study protocol conforms to the ethical guidelines of the 1975 Declaration of Helsinki (6th revision, 2008) as reflected in a priori approval by the institution's human research committee. The current study was derived from two projects approved in ethical committee of Golestan University of Medical Sciences (IR.GOUMS.REC.1401.563, IR.GOUMS.REC.1401.475).

**Table 1:** Demographic and clinic‐pathologic parameters in celiac and control groups.

Variables		Control	Celiac disease	*p*
Age		36.02 ± 1.79	40.90 ± 1.76	> 0.05^a^
Gender	Male	6 (13.3%)	10 (0.23.2%)	0.187^b^
	Female	39 (86.7%)	33 (0.76.8%)	
MARSH score	2 + 3a	—	17 (39.5%)	—
	3b + 3c		26 (60.5%)	
tTg‐IgA (unit/mL)		—	192.60 ± 30.06	—
Citrulline (nmol/mL)		11.37 ± 0.48	11.91 ± 0.41	0.68^a^
PTH (pg/mL)		300.1 ± 13.55	396.8 ± 7.33	< 0.0001^c^
Ca (mg/dL)		8.54 ± 0.33	9.66 ± 0.26	0.0097^c^
Vit D (ng/mL)		25.28 ± 2.118	27.43 ± 3.38	0.508^c^
HDL (mg/dL)		30 ± 1.74	33.53 ± 1.9	0.48^a^
LDL (mg/dL)		96.93 ± 4.88	99.54 ± 5.19	0.65^a^
Cholesterol (mg/dL)		149.9 ± 6.39	154.1 ± 5.5	0.49^a^
Triglyceride (mg/dL)		135.8 ± 13.93	112.7 ± 11.09	0.09^a^

^a^ Independent *t*‐test.

^b^ *χ* ^2^ test.

^c^ Mann–Whitney *U* test.

### 2.2 Biochemical Parameters Measurement

The levels of anti‐Ttg antibody (Table 1) and MARSH score were available from the previous study. Serum levels of the Dkk‐1 (Cat. Number: ZB‐10630C‐H9648, assay range: 5‐160 ng/ml) and sclerostin (Cat. Number: ZB‐13068C‐H9648, assay range: 7.5‐240 ng/ml) were measured using ELISA kits from ZellBio GmbH following manufacturer's instruction. Also, to measure PTH, we used an ELISA kit from ZellBio GmbH (Cat. Number: ZB‐11055C‐H9648, assay range: 60‐1920 pg/ml). Citrulline level was measured using ELISA kit from ZellBio GmbH (Cat. Number: ZB‐13718C‐H9648, assay range: 1.5‐48 nmol/ml). The levels of vitamin D3 and calcium were also measured using HPLC and spectrophotometry methods, respectively, in the Kavosh clinical laboratory, in Gorgan, Iran.

### 2.3 Data Analysis Methods

All values were expressed as mean ± standard error (SE). Using SPSS software (v.17), the Shapiro–Wilk test was performed to check data distribution. For qualitative variables, *χ* ^2^ test was used to compare case and control. The Mann–Whitney *U* or independent *t*‐tests were used to compare groups as indicated. Spearman's test was performed for correlation between variables. The *p* values of < 0.05 were considered statistically significant.

## 3 Results

### 3.1 Higher Bone Remodeling Factors: PTH and Calcium in Celiac Patients

As shown in Table 1, the patients were categorized according to the MARSH score of the duodenal biopsies (Scores 2 and 3a: normal/mild atrophy, Scores 3b, 3c: marked/complete atrophy in villi). In the current study, 39.5% of patients showed normal/mild villus atrophy, but 60.5% were in the category of marked/complete villus atrophy. The serum citrulline concentration was measured as a marker of reduced enterocyte mass [22] and we observed no significant difference between celiac patients and controls (Mann–Whitney test *p* = 0.6856).

Biochemical factor evaluation indicated that the lipid profile and vitamin D3 showed no significant difference between the two groups; however, the level of serum PTH was significantly (Mann–Whitney test *p* < 0.0001) higher in celiac patients (396.8 ± 7.33 pg/mL) compared to controls (300.1 ± 13.55 pg/mL). It should be noted that the used kit was for research use only and has detction range different from the clinical reference range of PTH; therefore the PTH data only was used for comparison between the groups. In addition, the level of serum calcium was significantly (Mann–Whitney test *p* = 0.0097) higher in celiac patients (9.66 ± 0.26 mg/dL) compared to controls (8.54 ± 0.33 mg/dL).

### 3.2 Elevated WNT Antagonists, Dkk‐1, and Sclerostin in Serum of Celiac Patients

**Figure 1:**
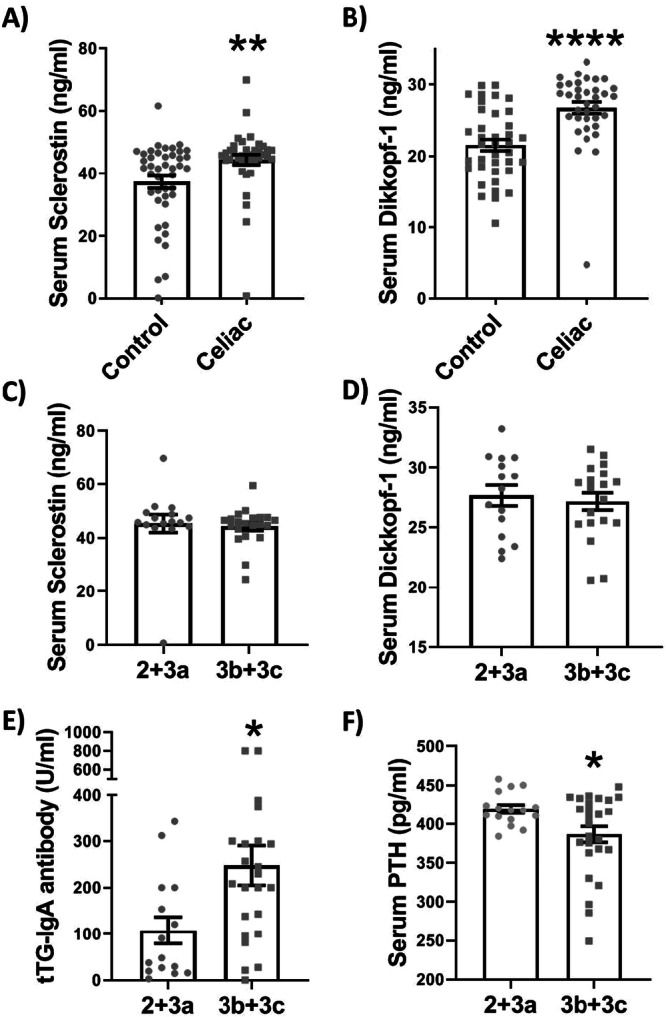
Serum levels of two WNT signaling antagonists; Dickkopf‐1 (A) and sclerostin (B) in celiac and control groups. Celiac patients categorized to two groups according the intestinal villus damage (MARSH score). MARSH score 2 + 3a indicates patients with no/mild atrophy and 3b + 3c represents patients with marked/complete atrophy. Serum sclerostin (C), Dickkopf‐1 (D), anti‐tissue‐transglutaminase IgA (tTG‐IgA) (E), and parathyroid hormone (PTH) (F) were compared between patients according to the intestinal villus damage. Each column represents mean ± standard error. **p = 0.0027, ****p < 0.0001, *p = 0.02.

We found significant (Mann–Whitney *U* test, *p* = 0.0027) upregulation of serum sclerostin levels (Figure 1A) in celiac patients (44.4 ± 1.6 ng/mL) compared to controls (37.2 ± 1.9 ng/mL). In addition, the level of serum Dkk‐1, another WNT signaling antagonist (Figure 1B) was also significantly (Mann–Whitney *U* test, *p* < 0.0001) higher in celiac patients (26.77 ± 0.8 ng/mL) compared to controls (21.55 ± 0.8 ng/mL).

For further evaluation, patients were categorized into two groups according to the intestinal villus atrophy (MARSH score). As indicated in Figure 1C,D, we found no difference regarding the Dkk‐1 and sclerostin levels between patients with no or mild villus atrophy (2 + 3a) compared to marked and complete villus atrophy (3b + 3c).

Other biochemical factors were also compared in the patients and interestingly we observed higher serum tTG‐IgA antibody (Student's *t*‐test, *p* = 0.02) and lower parathyroid hormone (Student's *t*‐test, *p* = 0.02) in patients with more severe intestinal damage (MARSH score 3b + 3c) than those with no or mild villus atrophy (Figure 1E,F).

### 3.3 Clinical Significance of Dkk‐1 and Sclerostin in Celiac Disease Diagnosis and Classification

To explore the importance of WNT antagonist changes in celiac disease, we analyzed the correlation of biochemical and clinical characteristics. Spearman's rank correlation revealed that both Dkk‐1 and sclerostin are correlated together (*R* = 0.6, *p* < 0.0001). Also, both showed a direct correlation with PTH (*R* = 0.69 for Dkk‐1 and *R* = 0.74 for sclerostin, *p* < 0.0001).

**Figure 2:**
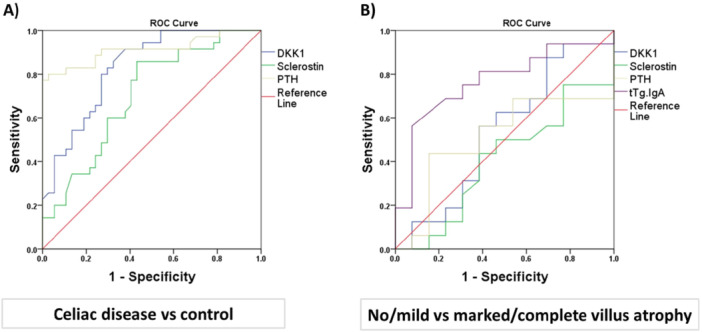
ROC curve analysis of Dkk‐1, sclerostin, and PTH in celiac disease (A) and classification of intestinal atrophy (B). The reference line is colored in red color.

Unexpectedly, there was no significant correlation between WNT antagonists and anti‐tissue‐transglutaminase IgA in the current study. However, the level of citrulline as a metabolite produced mainly by the enterocytes of the small intestine was also directly correlated with sclerostin (*R* = 0.71, *p* < 0.0001) and Dkk‐1 (*R* = 0.29, *p* = 0.012). On the other hand, PTH showed a direct correlation with calcium (*R* = 0.233, *p* = 0.04) and citrulline (*R* = 0.53, *p* < 0.0001). Altogether, the correlation analysis underscored the direct correlation between the WNT antagonists (Dkk‐1 and sclerostin), PTH, and citrulline as the enterocyte mass marker. For the next step, we evaluated the sensitivity and specificity of the factors correlated with enterocyte marker (citrulline) in the prediction of celiac disease and marked or complete intestinal villus atrophy (MARSH score 3b + 3c). As indicated in Figure 2A, the area under curve (AUC) of ROC curve analysis indicated that Dkk‐1 (AUC = 0.83, *p* < 0.0001), sclerostin (AUC = 0.7, *p* = 0.003), and PTH (AUC = 0.91, *p* < 0.0001) can successfully predict the celiac disease. The cutoff points for Dkk‐1 = 24.7 ng/mL with sensitivity = 80% and specificity = 73%, sclerostin = 44.7 ng/mL with sensitivity = 65% and specificity = 60% could potentially predict the celiac disease compared to the gold standard of an intestinal biopsy.

However, none of them can predict the MARSH score category in celiac disease except anti‐tissue‐transglutaminase IgA (AUC = 0.76, *p* = 0.01) with cutoff = 129.5 unit/mL able to discriminate patients with marked/complete intestinal atrophy from those with no/mild atrophy (sensitivity = 75% and specificity = 70%) (Figure 2B).

## 4 Discussion

Celiac disease is an enteropathy resulting from inappropriate adaptive immunity in response to digested gluten‐derived peptides. Intestinal epithelial barrier impairment plays a role in the pathogenesis of CD and patients are usually characterized by intestinal tissue damage including decreased enterocyte height, crypt hyperplasia, and villous atrophy [23].

The obtained results in this research show that serum levels of sclerostin, Dkk‐1, as bone negative regulators and WNT antagonists, and also the two other bone modulators, PTH and Ca, are significantly higher in celiac patients compared to control subjects.

The two WNT antagonists measured in the current study (sclerostin and Dkk‐1) function as potent suppressors of bone formation; however, there is evidence supporting their role in inflammatory disease indicating their immunomodulatory function. In a research conducted by Dhakad and colleagues, it was reported that serum sclerostin was significantly higher in rheumatoid arthritis (RA) patients compared to controls (*p* = 0.002); however, it was not correlated with bone mineral density (BMD) or disease activity [24]. Also, Ibrahim and colleagues reported that elevated sclerostin levels in RA patients contributed to joint damage and bone erosion in RA patients [25]. These results are in agreement with our observations of CD patients. On the other hand, Koch and colleagues found that Dkk‐1 contributes an important function in intestinal epithelial cell homeostasis and its depletion can promote intestinal recovery and repair in colitis, with hyperproliferation of epithelial cells and irregular crypt structure [14]. Kim and Choe found elevation of serum Dkk‐1 in patients who have Crohn's disease (*p* = 0.003) correlated directly with ESR, CRP, and pediatric Crohn's disease activity index [26].

The correlation between WNT signaling regulator genes and villus atrophy in celiac disease has been previously explored. Caliskan and colleagues reported that in the early stages of the celiac disease (MARSH scores 1–2), WNT signaling‐related genes LRP5 and CXADR genes, have high expression, but decrease in patients with villous atrophy. The expression of other WNT signaling genes including DVL2, CCND2, and NFATC1 showed lower expression in early stages but the highest expression in patients with villous atrophy (MARSH score 3b) [27].

Previous studies reported that the prevalence of primary hyperparathyroidism in celiac disease is higher than general population, suggesting a significant association between hyperparathyroidism and celiac disease [28].

Ganji and colleagues in a cross‐sectional study reported 16.4% of celiac patients have osteoporosis and 27.7% of them had high PTH levels which was significantly associated with osteoporosis in the femoral and spine [29].

Considering their common role in bone regulation, a cross‐talk between PTH and WNT antagonists has been postulated. Nagata and colleagues reported that PTH regulates circulating levels of sclerostin and FGF‐23 in a primary hyperparathyroidism model. Their results showed that patients with primary hyperparathyroidism have lower serum sclerostin levels than healthy controls, consistent with the idea of SOST downregulation by PTH [30].

The main characteristics of celiac disease are intestinal damage and villus atrophy which can be measured with MARSH scoring. Another quantitative marker of enterocyte mass is citrulline which has been reported to be lower in celiac disease and considered as a candidate marker of histopathological severity of damage [31]. However, in contrast, we observed no significant difference in citrulline levels in control and patient subjects. Similarly, research conducted by Douda and colleagues in 2023 reported that there were no statistically significant differences in the citrulline levels between the CD patients and the control group which is in agreement with our results [32].

Citrulline can also be produced from another mechanism through the function of endothelial nitric oxide synthase (eNOS), which can catalyze the production of NO and L‐citrulline from Arg [33]. Indeed, previous studies indicated that treatment of human umbilical cord vein endothelial cells with PTH reduces PTH receptor‐1, and upregulates eNOS mRNA and protein significantly. PTH treatment can also induce a significant increase in eNOS activity, as measured by the conversion of [(14)C]arginine to [(14)C] citrulline [34].

Although there is no direct correlation, however, the increased PTH, which is commonly observed in celiac patients, might indirectly affect L‐citrulline level through eNOS modulation. So maybe here in the current study, the citrulline level was affected by the metabolism of Arg induced by PTH‐stimulated increase in the eNOS activity rather than enterocyte integrity in CD. Here in our study, we also found a direct correlation between PTH and citrulline (*R* = 0.53, *p* < 0.0001).

Calcium malabsorption is a well‐known characteristic of unmanaged celiac disease [35]; however, we found elevated calcium levels in CD which was positively correlated with PTH. It is widely recognized that in response to PTH, the concentration of calcium in circulation will be raised; the positive association we observed could be related to the function of PTH in the positive regulation of serum Ca.

Here in the current study, we were going to evaluate the correlation between intestinal atrophy and WNT antagonist, then the samples without intestinal biopsy were not included which could be considered as limitation for sample size. Indeed, the BMD and bone remodeling disease like osteoporosis may affect the level of both WNT antagonists, sclerostin [36] and DKK‐1 [37]. In the current study, although we measured the Ca and PTH levels, but the BMD of participants was unknown which could be considered as a limiting factor.

## 5 Conclusion

Our study showed that elevation of the two WNT signaling antagonists, Dkk‐1 and sclerostin, along with PTH as another bone modulator in the serum of celiac patients correlated directly with the level of citrulline, a potential marker of enterocyte mass. These findings altogether suggest the importance of Dkk‐1 and sclerostin in the pathogenesis of celiac disease but, more studies are appealing to investigate their potential diagnostic and therapeutic application in the future.

## Author Contributions

Mohammed Nibras Thabit Al‐Ani and Saad Kadhim Taher Yahya contributed in experiments and data collection equally, Majid Dastorani helped in data analysis and data collection, Hanie Mirkarimi and Sima Besharat helped in sample collection and Marie Saghaeian Jazi contributed in project design and data analysis. All authors contributed in manuscript drafting.

## Conflicts of Interest

The authors declare no conflicts of interest.

## Supporting information

Supporting File

## Data Availability

Data sharing is not applicable to this article as no data sets were generated or analyzed during the current study.
